# Comparison of methods to experimentally induce opacification and elasticity change in ex vivo porcine lenses

**DOI:** 10.1038/s41598-021-02851-6

**Published:** 2021-12-03

**Authors:** Manuel Ruiss, Martin Kronschläger, Andreas Schlatter, Thomas Dechat, Oliver Findl

**Affiliations:** 1 Department of Ophthalmology, Hanusch Hospital, Vienna Institute for Research in Ocular Surgery (VIROS), a Karl Landsteiner Institute, Heinrich-Collin-Straße 30, 1140 Vienna, Austria; 2 1st Medical Department, Hanusch Hospital, Ludwig Boltzmann Institute of Osteology at Hanusch Hospital of OEGK and AUVA Trauma Centre Meidling, 1140 Vienna, Austria

**Keywords:** Lens diseases, Experimental models of disease

## Abstract

At the moment, cataract, which is the opacification of the eye’s lens, can only be treated by surgery. In order to develop and test new pharmacological treatment strategies for the disease, there is a need for an appropriate in vitro model using ex vivo animal lenses. In this study, porcine lenses were incubated in either culture medium, glucose, triamcinolone acetonide, sodium chloride, hydrogen peroxide, sodium selenite, neutral buffered formalin, or were exposed to microwave heating to experimentally induce lens opacification. Changes in the lens morphology, weight, size, and elasticity were monitored 7 days after treatment. The fastest induction of dense opacification was seen in lenses exposed to sodium chloride, neutral buffered formalin, and microwave heating. No change in the size and weight of the lenses were detected, whereas loss in elasticity could be detected in lenses treated with formalin solution or microwave heating. Thus, neutral buffered formalin- and microwave-treated ex vivo porcine lenses seem to be a suitable model for mature cataracts, whereas hypertonic sodium chloride may be useful for studies on osmolarity-induced lens opacification.

## Introduction

Cataract, the opacification of the eye’s lens, is the number one reason for blindness worldwide^1^. At the moment the disease can only be cured by surgery, while there is no approved pharmacological agent for its therapy or prevention. In order to develop and test new pharmacological candidate drugs for cataracts, there is a need for an appropriate, fast, cheap, and simple model of lens opacification.

In laboratory animals, there are different methods to induce cataract in vivo, such as subcutaneous or intraperitoneal injection of sodium selenite (Na_2_SeO_3_)^2^, dietary streptozotocin or galactose^3^, dietary naphthalene^4^, intraperitoneal injection of l-buthionine-(S,R)-sulfoximine (BSO)^5^, ultraviolet radiation exposure^6^, or hyperbaric oxygen treatment^7^. Furthermore, inherited transgenic or knockout animal models of cataract are available^8^. However, an animal laboratory unit is not accessible for every researcher and experiments on laboratory animals can be time-consuming and expensive. Furthermore, postmortem human eyes are not readily available.

An alternative approach is the use of ex vivo animal lenses for cataract induction in vitro. According to a recent review about the pharmacotherapy of cataracts, the most widely used candidate substances to induce lens opacification in ex vivo lenses are Na_2_SeO_3_, hydrogen peroxide (H_2_O_2_), and glucose^9^. Other models of cataract formation successfully used in cataract wet labs include injection of formaldehyde into lenses, microwave heating of the eye globes, or bathing of lenses in hypertonic sodium chloride (NaCl) solution^10–16^. Furthermore, clinical studies have shown that lens opacification is one of the side effects of intravitreal triamcinolone acetonide injections and extended use of steroids by any route (e.g. intraocularly, topically, or systemically)^17–21^.

During the aging process, starting at about the age of 40 years, conformational changes of proteins lead to their accumulation in the lens. This results in an increasing loss of elasticity and, hence, progressive hardening of the lens as well as increased light-scatter and loss of lens transparency. The clinical correlate of this phenomenon is the onset of presbyopia and the formation of cataract^22^.

Since, to our knowledge, there is no report that compares the potential of the above-mentioned methods to induce lens opacification and lens elasticity change in ex vivo porcine lenses, the aim of this study is to test the efficacy of these different approaches to experimentally induce cataract in vitro.

## Results

**Figure 1:**
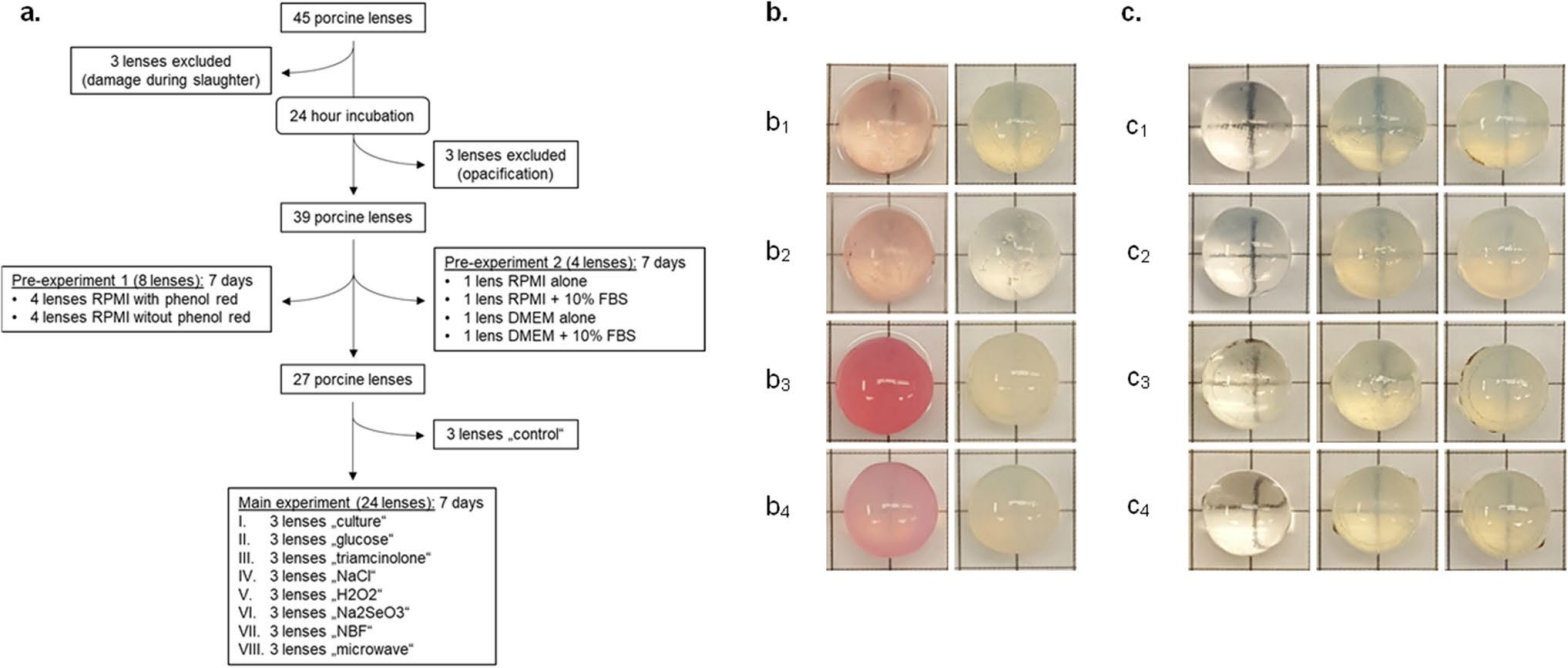
Flow chart of porcine lenses used for the different experiments and the effect of phenol red, different culture media, and fetal bovine serum (FBS) on them. (**a**) Flow chart depicting the number of porcine lenses used in the pre- and the main experiments. (**b**) Four porcine lenses treated with RPMI medium containing phenol red (left) and 4 lenses treated with RPMI medium without phenol red (right). Lenses were photographed 7 days after incubation in culture medium only (**b**_**1**_), triamcinolone acetonide (**b**_**2**_), NaCl (**b**_**3**_), or H_2_O_2_ (**b**_**4**_). In all cases, RPMI medium was supplemented with penicillin and streptomycin. (**c**) Porcine lenses were incubated in either RPMI medium alone (**c**_**1**_), RPMI medium with 10% FBS (**c**_**2**_), DMEM alone (**c**_**3**_), or DMEM with 10% FBS (**c**_**4**_). Photographs were taken after an incubation period of 0 days (left), 4 days (middle), and 7 days (right). In all cases, culture medium was supplemented with penicillin and streptomycin.

A total of 45 porcine lenses were used for this study, of which 6 lenses had to be excluded since 3 lenses developed opacification after the 24-h incubation period and 3 eyes were excluded due to damage induced during the slaughter process (Fig. 1a).

Before starting with the main experiments, different culture conditions were tested. First, 4 porcine lenses were incubated in either RPMI medium with or without phenol red. Incubation of lenses in medium supplemented with phenol red for 7 days resulted in lenses incorporating the pink color (Fig. 1a,b). Second, since we have achieved good results with RPMI medium in culturing lens epithelial cells in previous studies, and since DMEM is one of the most used media in cell culture experiments, we wanted to compare the influence of both media on lens transparency. Additionally, since fetal bovine serum (FBS) is used in most experiments on ex vivo lenses for the 24-h incubation period, we also assessed the influence of FBS on lens opacification-induction. We did not find any differences between RPMI medium or DMEM with or without FBS concerning lens opacification after the 7-day period (Fig. 1c). Hence, for further experiments, RPMI medium supplemented with penicillin and streptomycin but without phenol red was used and FBS was only added for the 24-h incubation period.

### Lens morphology and opacification

No differences in opacification gradings between the two masked examiners (all groups P > 0.500) and between photographs of lenses that were completely immersed in culture medium or those without culture medium in the well (P > 0.300) were found.

**Figure 2:**
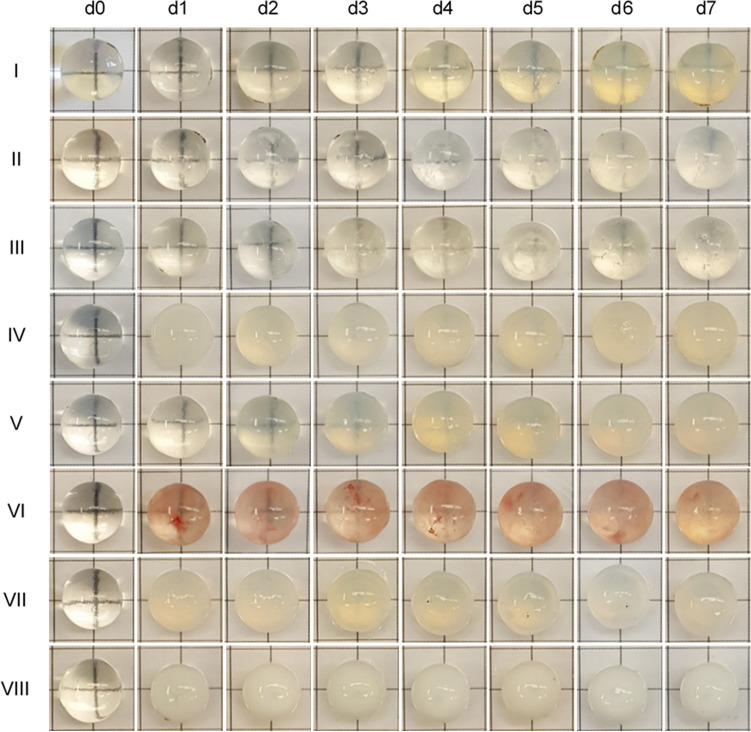
Progression of opacification over time in lenses treated with different methods to induce lens clouding. d = day; I–VIII = groups I to VIII.

None of the porcine lenses used in the experiments of this study showed an opacification after the 24-h incubation time. Porcine lenses of group I (culture medium only) developed grade 1 opacification about 2 days after incubation, progressing to grade 2 on days 4 to 5. Two out of three lenses in group II (glucose) and group VI (Na_2_SeO_3_) showed grade 2 and one lens in both groups showed grade 3 opacification after the 1-week period. However, a pink coloration was detected in lenses of the latter group starting at day 1 after incubation. No significant difference was found in lens opacification grading between group I and groups II and VI (both P > 0.050) after 7 days. All the lenses of the other groups developed grade 3 clouding, however, with different onsets. In group III (triamcinolone) and group V (H_2_O_2_) dense opacification appeared between days 5 to 6 and on day 4, respectively, whereas in groups IV (NaCl), VII (neutral buffered formalin, NBF), and VIII (microwave) it was immediately seen on day 1. Significant differences in lens clouding were found between group I and groups III, V, IV, VII (all P = 0.008), and VIII (P = 0.009) after 1 week of incubation. Furthermore, changes in opacification gradings over time were found within all groups (Fig. 2, Table 1).

**Table 1:** Grading of porcine lens opacification with different treatment conditions over time.

Group	n_total_	Degree of opacification								n_observed_	P_within_
		d0	d1	d2	d3	d4	d5	d6	d7		
I	3	0	0	1	1	2	2	2	2	2	0.005
		0	0	1	1	1	2	2	2	1	
II	3	0	1	2	2	2	2	2	2	1	0.019
		0	1	1	2	1	2	2	2	1	
		0	1	1	1	2	2	3	3	1	
III	3	0	1	2	2	2	2	3	3	2	0.005
		0	1	2	2	2	3	3	3	1	
IV	3	0	3	3	3	3	3	3	3	3	0.004
V	3	0	1	2	2	3	3	3	3	2	0.004
		0	2	2	2	3	3	3	3	1	
VI	3	0	1	2	2	3	3	3	3	1	0.019
		0	1	2	2	2	2	2	2	1	
		0	2	2	2	2	2	3	2	1	
VII	3	0	3	3	3	3	3	3	3	3	0.004
VIII	3	0	3	3	3	3	3	3	3	3	0.004

Group I: cell culture medium only, group II: glucose, group III: triamcinolone acetonide, group IV: NaCl, group V: H_2_O_2_, group VI: Na_2_SeO_3_, group VII: neutral buffered formalin, VIII: microwave. n = number; n_total_ = total number of lenses per group; n_observed_ = number of opacification grading patterns seen per group; d = day; P_within_ = P value within group. Statistical significance within the groups was analysed using Friedman’s multiple comparison. P < 0.05 was considered significant.

**Figure 3:**
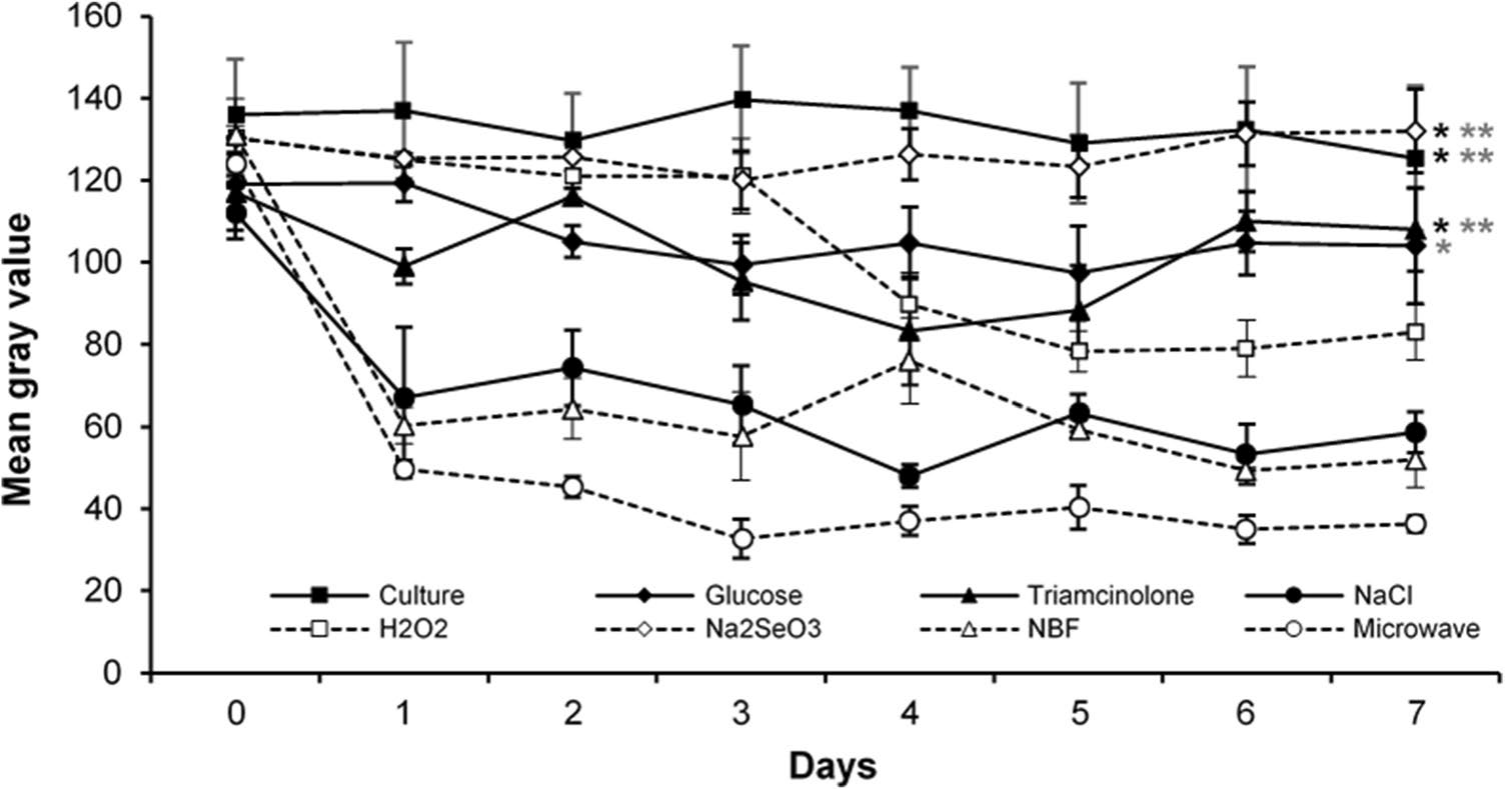
Change in mean gray value of the porcine lenses in the different treatment groups over time. Black asterisk (*) indicates significant differences between group VII (NBF) and the respective groups, gray asterisk (*) indicates significant differences between group VIII (microwave) and the respective groups. n = 3 porcine lenses per group. Data are depicted as mean ± standard error of the mean (s.e.m.). Statistical significance between the groups was analysed using Kruskal–Wallis-test. A P value < 0.05 was considered significant; *P < 0.05, **P < 0.01.

Significant differences in mean gray value were seen between the groups starting from day 1 to day 7 after incubation (at all days P < 0.05). The mean absolute error (MAE) of the mean gray value between day 0 and day 7 for the different treatment groups was 13 ± 19 (group I), 31 ± 24 (group II), 32 ± 28 (group III), 53 ± 32 (group IV), 51 ± 40 (group V), 21 ± 40 (group VI), 79 ± 23 (group VII), 88 ± 7 (group VIII). One week after exposure to the opacification-inducing methods, the mean gray value differed between lenses of group VII and groups I (P = 0.040), III (P = 0.040), and VI (P = 0.015), as well as between group VIII and groups I (P = 0.006), II (P = 0.024), III (P = 0.009), and VI (P = 0.003). Within groups a significant difference in mean gray value was detected for NaCl- (P = 0.038), H_2_O_2_- (P = 0.022), NBF- (P = 0.002), and microwave-treated (P < 0.0001) porcine lenses over time (Fig. 3).

### Lens size and weight

**Figure 4:**
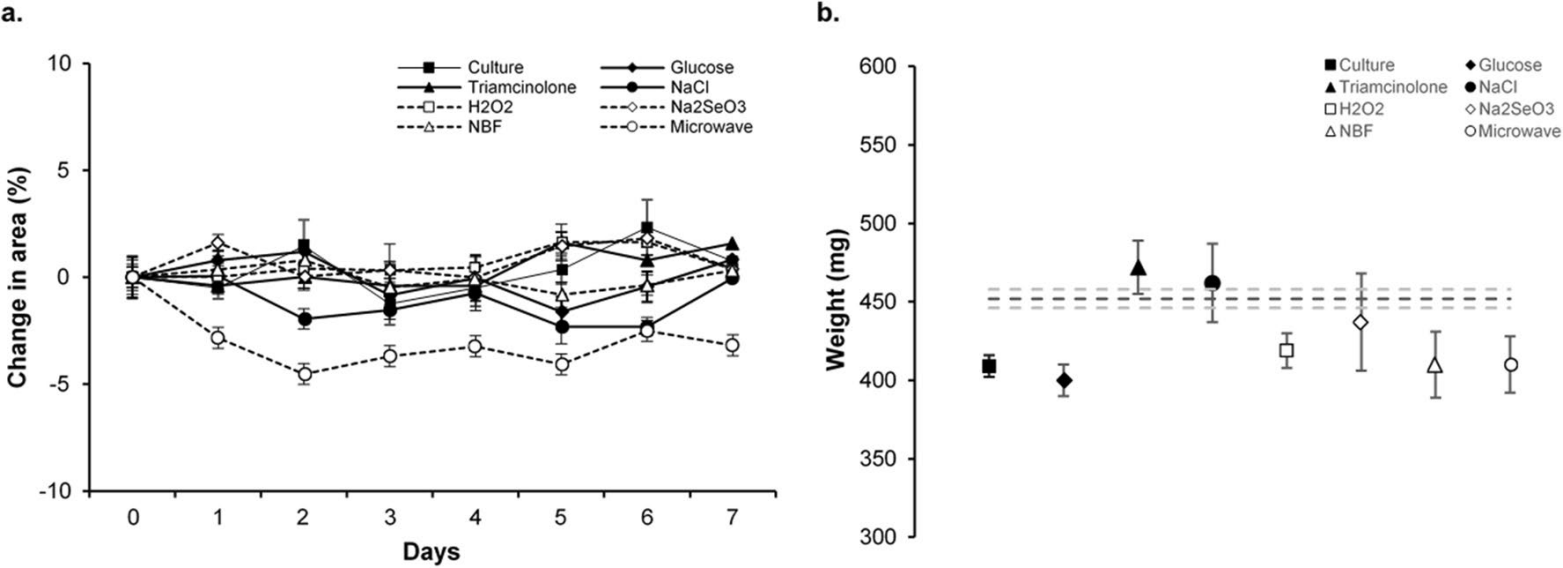
Change in image area over time and in wet weight of porcine lenses in the different treatment groups. (**a**) Change in area of lenses in groups I to VIII between day 0 and day 7. (**b**) Change in wet weight of lenses in groups I to VIII at day 7. Dotted lines represent mean wet weight (dark gray) and standard error of the mean (s.e.m., light gray) of lenses directly weighed after dissection (control). n = 3 porcine lenses per group. Data are depicted as mean ± standard error of the mean (s.e.m.).

Change in porcine lens area between day 0 and day 7 was 0.8 ± 4.5% (median − 1.2, range − 2.5 to 5.9) in group I, 0.8 ± 2.0% (median 1.2, range − 1.3 to 2.7) in group II, 1.6 ± 0.7% (median 1.2, range 1.2 to 2.4) in group III, 0 ± 1.2% (median 0.3, range − 1.4 to 1.0) in group IV, 0.4 ± 0.7% (median 0, range 0 to 1.1) in group V, 0.4 ± 0.7% (median 0, range 0 to 1.2) in group VI, 0.3 ± 1.3% (median 1.1, range: − 1.2 to 1.1) in group VII, and − 3.2 ± 3.1% (median − 3.5, range − 6.1 to 0.1) in group VIII. However, no differences in lens size were detected between or within the groups during the 1-week incubation period (in all cases P > 0.100) (Fig. 4a).

The wet weight of the 3 porcine lenses measured directly after dissection (control) was 452 ± 10 mg (median 451, range 442 to 463). One week after incubation wet weight was 409 ± 11 mg (median 405, range 400 to 422) in group I, 400 ± 17 mg (median 404, range 381 to 415) in group II, 472 ± 29 mg (median 487, range 438 to 490) in group III, 462 ± 43 mg (median 447, range 428 to 510) in group IV, 419 ± 19 mg (median 424, range 398 to 436) in group V, 437 ± 54 mg (median 432, range 385 to 493) in group VI, 410 ± 36 mg (median 392, range 386 to 451) in group VII, and 410 ± 30 mg (median 415, range 377 to 437) in group VIII. No significant differences in weight between lenses that were directly weighed after dissection compared to lenses of groups I to VIII measured at day 7 were detected (P = 0.086) (Fig. 4b).

### Lens elasticity

**Figure 5:**
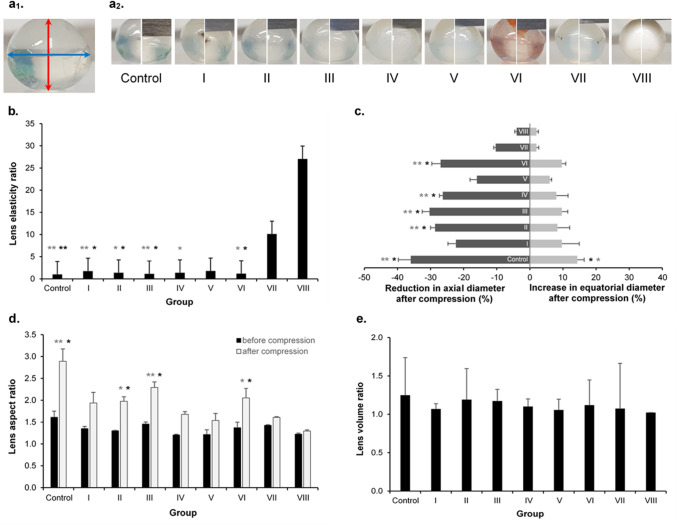
Effect of different treatment methods on porcine lens elasticity. (**a**) (**a**_**1**_) Image analysis was used to determine axial diameter (red arrow) and equatorial diameter (blue arrow). (**a**_**2**_) One example of a porcine lens before (left) and after (right) compression for the control and each group is shown. (**b**) The lens elasticity ratio (weight/height) after lens compression is shown for each treatment condition and was calculated as the ratio to the mean in the control group. (**c**) Reduction in axial diameter and increase in equatorial diameter after compression is shown for the control group and each opacification-inducing method. (**d**) Lens aspect ratio before and after compression was calculated by dividing the equatorial diameter by the axial diameter for each group and the control. (**e**) Lens volume ratio was calculated by dividing the pre-compression by the post-compression volume for each group and the control. (**b**–**e**) n = 3 porcine lenses per group. Results are expressed as mean ± standard error of the mean (s.e.m.). Kruskal–Wallis-test was used for statistical analysis in (**b**), (**d**), and (**e**), and One-Way ANOVA with Tukey’s post hoc test in (**c**). Black asterisk (*) indicates significant differences between group VII and the other respective groups, gray asterisks (*) indicate significant differences between group VIII and the other respective groups. A P value < 0.05 was considered statistically significant; *P < 0.05, **P < 0.01.

Lens elasticity ratio (change in weight over height) was determined using the heigh gauge/electronic balance approach. After compression, it was shown that lenses exposed to NBF or microwave heating for 7 days were harder than lenses of the control group (P = 0.004 and P = 0.001, respectively). Further differences were found between group VII and groups I (P = 0.020), II (P = 0.027), III (P = 0.020), and VI (P = 0.032), as well as between group VIII and groups I (P = 0.009), II (P = 0.012), III (P = 0.009), IV (P = 0.032), and VI (P = 0.015) (Fig. 5b).

Similar results were found when the change in axial diameter before and after compression was determined using image analysis. Significant differences were found when group VII and group VIII were compared to groups II (P = 0.012 and P = 0.002, respectively), III (P = 0.027 and P = 0.003, respectively), IV (P = 0.028 and P = 0.003, respectively), and VI (P = 0.040 and P = 0.004, respectively). Additionally, there was a significant difference in the change of axial diameter between control lenses and those exposed to NBF and microwave heating (P = 0.015 and P = 0.002, respectively) and the same was seen for change in equatorial diameter (control vs. VII: P = 0.018; control vs. VIII: P = 0.029) (Fig. 5c).

No significant differences were found in lens volume between (before compression: P = 0.110, after compression: P = 0.130) and within (all groups P > 0.100) groups and lens aspect ratio before compression (P = 0.056). However, after applying pressure onto the lenses, differences in the lens aspect ratio were found between group VII and groups II (P = 0.045), III (P = 0.037), and VI (P = 0.047) as well as between group VIII and groups II (P = 0.027), III (P = 0.004), and VI (P = 0.017). Additionally, the lens aspect ratio differed between control lenses and those exposed to NBF and microwave heating (P = 0.012 and P = 0.002, respectively) (Fig. 5d,e).

## Discussion

Porcine eyes are widely used in ophthalmological research and cataract wet labs since they share some similarities in their morphology with the human eye^23^. Furthermore, as opposed to postmortem human eyes or laboratory animals, pig eyes are readily available. Therefore, porcine lenses would be an ideal in vitro model to induce experimental cataracts in order to develop and test new pharmacological strategies to treat or prevent the disease. This is, to our knowledge, the first study that compares different methods to induce lens opacification and analyses lens elasticity in ex vivo pig lenses.

In our experiments, porcine lenses were cultured in medium containing no phenol red, because in some of the lenses phenol red had accumulated over the 7-day period. Since we did not observe any differences between DMEM and RPMI medium regarding the temporal onset and grade of lens opacification, all of our experiments were performed using RPMI medium. Additionally, no difference in the clouding of the lenses was found when the culture medium was supplemented with or without 10% FBS. An incubation period of 24 h was used in this study before starting with the actual experiments to discriminate between damaged and undamaged lenses. Although in some studies on ex vivo lenses an incubation period of only 2 h is used, we believe that a longer incubation period would be more suitable to avoid using damaged lenses because transparency was detected in some damaged lenses even after 24 h of incubation^24^. Porcine lenses incubated in RPMI medium alone developed grade 1 opacification on days 2 to 3, which progressed to grade 2 opacification on days 4 to 5. Contrary, Wang et al. reported that in their experiments, porcine lenses started to lose transparency after 8 h in medium without serum, whereas clarity was maintained for up to 5 to 6 days with 1% pig serum^25^. However, they have used a different culture medium (TC-199) and further studies are necessary to evaluate the influence of pig versus bovine serum on lens opacification induction.

The addition of culture medium supplemented with high concentrated glucose to ex vivo lenses is a widely used in vitro model of diabetic cataract. The underlying mechanism is multifactorial and seems to involve osmotic and/or oxidative changes of the lens via the aldose reductase/polyol pathway^26^. Under our culture conditions, grade 1 opacification developed at day 1 and progressed to grade 2 on days 2 to 3 as well as grade 3 on days 6 to 7. In contrary, in goat lenses glucose-induced lens clouding also started early (after 8 h) but complete clouding of the lens was already detected after 72 h^27–29^. Furthermore, in Sprague–Dawley rats, high glucose-induced opaque rings appeared in most lenses during the 5-day incubation period^30^. In all of these experiments glucose concentrations of 50–55 mM were used, hence, the difference in opacification onset between our results and the mentioned literature reports cannot be explained by a concentration difference. Rather, the differences might be explained by the use of different animal lenses (goat, rat) or different culture media (artificial aqueous humour, M-199 media).

Intravitreal injection of triamcinolone acetonide is used in ophthalmology for the treatment of retinal diseases like macular edema. As a side effect of the therapy, cataract formation may arise not only after injection of higher doses (20 or 25 mg) but also with the standard concentration of 4 mg triamcinolone acetonide^17–20,31,32^. It is thought that the reason for steroid-induced cataract may be oxidative stress within the lens^33^. In Brown-Norway rats with vitamin E deficient chow, instillation of 1% prednisolone acetate solution into the eyes led to cortical cataracts and an opacified subcapsular layer in 91.7% of rats after 15 months^34^. On the other hand, in lenses of Sprague–Dawley rats incubated with 5 μM dexamethasone, cataract developed after 3 days and progressed to a full cataract by day 7^33^. This last study is in agreement with our in vitro model on porcine lenses treated with triamcinolone acetonide since we’ve detected opacification after day 2, which progressed to grade 3 from day 5 to 7. However, faster cataract induction may occur with higher concentrations of triamcinolone or when using other steroids.

Hypertonic NaCl may produce osmotic disturbances in the lens via disruption of its water/ion balance and subsequently cataract formation. We found induction of grade 3 lens opacification immediately after 1 day incubation in 5% NaCl solution. Similarly, in C57BL/6 mice it was shown that hypertonic eye drops (500 and 1000 mOsmol/kg) disrupted lens transparency in a stronger manner than isotonic (300 mOsmol/kg), or hypotonic (100 mOsmol/kg) NaCl solution^35^. Furthermore, in porcine eyes lens clouding was induced 45 min after incubation of the pig eyeballs in hypertonic NaCl (9%, 19%, and 31.5%), whereas lenses incubated in normal saline remained transparent^16^.

Elevated levels of H_2_O_2_ are characteristic for cataractous lenses and human age-related cataracts^36^, hence, it is widely used as an oxidative stress-induced cataract model. For example, H_2_O_2_ was able to induce cataracts in lenses of Sprague–Dawley rats after 24 h^36–38^, in lenses of Wistar rats after 1 h^39^, in goat lenses after 33 h^40^, in New Zealand White Rabbits after 24 h^41^, and in C57BL/6 mice after 4–5 days^42^. This difference in the onset of lens opacification might be explained by the different concentrations of H_2_O_2_ (100 μM to 10 mM) used in these experiments or due to an animal-specific effect. We found grade 1 to grade 2 opacification after 24 h of incubation in 10 mM H_2_O_2_, which progressed to grade 3 after 3 days of incubation. Moon et al. found induction of pig lens clouding in the first 6 h after incubation with 0.2 mM H_2_O_2_ and the first hour after incubation with 0.5 mM H_2_O_2_. However, with both concentrations, the nuclear regions remained clear during the 24-h study period^43^. Further, Wang et al. reported that H_2_O_2_ less than 1.5 mM had very little impact on lens transparency in porcine lenses, while with higher than 5 mM concentrations severe cortical cataracts were detected within 24 h. Additionally, they found that under the same culture conditions lens opacification was easier induced in rat than in porcine lenses^25^.

Another widely used in vitro model for oxidative stress-induced lens opacification is the use of Na_2_SeO_3_. It was reported that 100 μM to 200 μM of the substance led to grade III opacification in Wistar rats^44–49^ and Sprague–Dawley rats^50,51^ after a 24-h incubation period. In our porcine model, 200 μM of Na_2_SeO_3_ induced grade 1 or grade 2 cataract after 1 day of incubation, which progressed to grade 3 starting on day 4. However, all lenses incubated with sodium selenite under our culture conditions showed a pink/reddish coloration after 24 h of incubation. The underlying mechanism of this phenomenon is unclear. We speculate, that the coloration of the lenses is due to the reducing effect of glutathione (GSH), which is found in high concentrations in the lens^52^. It was shown that GSH is able to reduce sodium selenite via selenium intermediates into red elemental selenium (Se^0^)^53^. None of the above-mentioned studies on rats report on lens color changes induced by Na_2_SeO_3_, although, a slight pink complexion of the rat lenses after incubation in Na_2_SeO_3_ can be observed in Geraldine et al.^44^ GSH content may differ between species and this might explain the differences seen in lens coloration. Furthermore, the coloration of the lenses might occur due to the interaction of Na_2_SeO_3_ with components of the culture medium. Further studies are necessary to analyse this phenomenon.

NBF acts by denaturing proteins causing the lens to become stiffer and to opacify. Injection of formalin into lenses of goats^11,13^, pigs^10,14,54^, or postmortem humans^12,55^ either alone or in combination with other substances (e.g., glutaraldehyde, methanol, ethanol, or propanol) at different concentrations (20% to 38%) have been proposed as models for cataract wet labs. We have used NBF in a concentration of 10% in our experiments since Oakey et al. found that soaking porcine lenses in this concentration of NBF for 2 h followed by 24-h incubation in BSS simulated human cataractous lens hardness^56^. With 10% NBF hardening of the lens and an opacification grade 3 could be induced after 24 h. Very dense and hard cataracts were also described in all of the above-mentioned reports. However, a direct comparison is difficult since in the other studies formalin was injected into the lenses while we have bathed them in NBF.

Another method often used for cataract wet labs is the induction of cataracts by microwave heating. For example, lens opacification, which resembled human mature cataract, was induced in porcine eyes after heating them in a 700 W microwave oven for 9 s and additional 4 s at full power^15^. In a similar experiment, Machuk et al. found that 6 to 10 s microwave heating of porcine lenses led to higher opacity than in lenses treated with 37% formalin^54^. Furthermore, Shentu et al. found that in a pre-heated microwave oven lens protein degeneration and nuclear hardening were achieved after 5–10 s, however, best results were yielded with a combination of fixative treatment and microwave heating^57^. All of these studies were done in whole eyes of pigs, whereas we have used microwave heating on dissected porcine lenses. In a non-pre-heated 800 watts microwave oven using full power it needed 15 to 20 s to induce white cataract and lens hardening in our model. It was necessary to fully immerse the lenses in culture medium while heating to achieve uniformly lens opacification. We found that microwave heating induced the densest cataract of all groups. Age-related cataracts arise when the order of the crystallins get disturbed leading to light-scatter, progressive hardening of the nucleus, and a gray-white opacity^22^. Similar, a change in lens elasticity and a white opacity was also detected in our porcine lenses treated with NBF or microwave heating. However, in further studies it would be interesting to analyze if the protein structure changes induced by formalin or microwave are comparable to those of naturally developing cataracts.

We could not observe a specific opacification development towards nuclear, cortical, or posterior subcapsular cataracts with our induction methods of acute cataract. This might be different when lens opacification would be induced by subthreshold stress. A pharmacological in vitro screening method should be fast and robust, hence, subthreshold models might not be suitable for this reason since they are more time-consuming and consequently more prone to bias.

Changes in size and weight of lenses (“swelling”) incubated under osmotic conditions were reported in the literature. For example, 48-h incubation of Sprague–Dawley rat lenses in 0.5 mM H_2_O_2_ led to a 31% lens weight gain, after which it plateaued^36^. Additionally, 24-h incubation of C57BL/6 mice lenses in the same concentration of H_2_O_2_ led to a 13% increase in lens weight^42^. Furthermore, bathing of Sprague–Dawley rat lenses in 0.2 mM Na_2_SeO_3_ and goat lenses in 10 mM H_2_O_2_ led to an increase in lens weight after 24 h and 72 h, respectively^51,58^. On the other hand, in porcine lenses only a small degree of hydration (7.5% increase in wet weight after 48-h incubation) was observed with 1.5 mM H_2_O_2_^25^. No significant changes in image area size and weight of the porcine lenses in the different groups was found in our study. In our model, the weight of the lenses in the different groups was measured solely at the end of the 7 days and compared to lenses weighed directly after the dissection. This was done in order to not manipulate the lenses and to prevent damage to lenses or lens capsules. Hence, we cannot exclude that time-dependent fluctuations in weight might have been overseen in our model. Furthermore, Sugiura et al. reported that the capsule of porcine lenses is thicker than those of humans^10^ and this may also be true for capsules of other animals. Hence, the thicker capsule may act as a stronger barrier against osmotic changes. Additionally, Hernebring et al. found that H_2_O_2_-induced lens swelling occurs initially in cataractogenesis of rats but rather late in mice, which is a further argument for animal-specific differences^42^. On the other hand, as shown in our pre-experiments, phenol red was easily incorporated into the lenses, especially when they were treated with NaCl and H_2_O_2_ (see Fig. 1b_3_,b_4_).

The primary aim of this study was to experimentally induce cataract in an in vitro approach using ex vivo porcine lenses. Nuclear cataracts are not only characterized by a grey-white opacification but also by increasing lens stiffness with age^22^. To our knowledge, there are no studies that incorporate lens elasticity measurements in studies about cataract induction in ex vivo animal lenses in vitro. For beginning cataract surgeons, it is important to practice the surgery under a situation that resembles real life, which includes using hard lenses in cataract wet labs. This is important to learn that more phacoemulsification energy is necessary to operate harder nuclei and to improve one’s skills to avoid complications like macular edema or corneal decompensation. Hence, in studies that aimed to develop suitable cataract models for wet labs, analysis of the nuclear hardness was a substantial part. In these studies, it was found that exposing lenses to formalin injections^10–14,55,57^ or microwave heating^15,57^ increased lens stiffening, whereas lenses exposed to NaCl bathing still demonstrated a soft nucleus after the treatment^16^. This is in conjunction with our findings. Only microwave heating and NBF-treatment led to a hardening of the lenses, whereas NaCl incubation left the lens elasticity rather unchanged. This might be due to the possible different underlying mechanisms of opacification-induction. As mentioned above, glucose, triamcinolone acetate, NaCl, H_2_O_2_, and Na_2_SeO_3_ induce lens opacification via oxidative and/or osmotic stress. On the cellular level, both mechanisms together or alone may induce peroxidation of phospholipids in the lens epithelial cell (LEC) membrane, disturbances of the ion microcirculation in the lens membrane (e.g. loss of Ca^2+^-ATPase or Na^+^/K^+^-ATPase, calcium increase) as well as oxidation of lenticular proteins. This results in lens crystallin proteolysis and their subsequent transformation into insoluble protein aggregates^21,34,39,44,47^. The endpoint of both mechanisms is apoptosis of LECs leading to disturbance of lens homeostasis and consequently to lens opacification^26,31,32,41^. Contrary, formalin and microwave heating act by directly cross-linking and degrading proteins with immediate induction of nuclear hardening, lens cell death, and faster induction of lens opacification^10,11,57^. Lens hardness might play an important role for in vitro screenings of pharmacological drugs in the treatment of cataracts. Whereas an agent for prophylaxis of cataracts should ideally be best applied before the occurrence of lens opacification, a pharmacological candidate substance for treating the disease should not only be able to reverse the beginning but also more pronounced stages of the disease. Hence, when screening for a potential pharmaceutical agent to treat cataracts, it should be taken into account that age-related changes in lens capsule elasticity and nuclear hardness might influence the penetration of the drug into the lens. Moreover, efficacy of the candidate drug could be dependent on the degree of cataract expression. Therefore, staging of cataract and assessing the health of the tissue including microscopic and biochemical methods (e.g. LEC and lens fiber morphology, apoptosis, ATP assays, concentration of antioxidant components) might be essential. We have employed the method described by Tsuneyoshi et al. as well as the image analysis model described by Cheng et al. for lens elasticity determination^59,60^. Although, the first method is skill-dependent concerning the critical step of lowering the height gauge onto the lens surface without destroying the lens, both methods yielded comparable results in our porcine lens model.

In conclusion, the fastest lens opacification was induced when using microwave heating as well as incubation of lenses in NBF or hypertonic NaCl. However, loss of elasticity, as described for human age-related cataracts, was only seen with microwave heating and incubation in NBF. Hence, microwave- and NBF-treated lenses may be the most suitable models for mature cataracts, whereas NaCl might be used for osmotically-induced cataracts in porcine lenses.

## Methods

Porcine eyes were purchased from a local butcher and, in order to avoid lens damage or opacification, were dissected before boiling water or hot steam was used to remove pig’s hair. The eyes were immediately transferred to the laboratory in a thermal bag and were kept in a refrigerator at a temperature of 4 °C until further processing. Dissection of the eyes was performed 4 to 6 h after the pick-up from the butcher. No differences in lens morphology were detected concerning the time between collection and procession of the eyes.

To remove the lenses, the porcine eyes were entered through the sclera using scissors, followed by gentle removal of the vitreous and cutting through the zonules. After dissection, one lens was immediately transferred in one well of a 12-well cell culture plate (Thermo Fisher Scientific Inc., Roskilde, Denmark) and was incubated in Roswell Park Memorial Institute (RPMI) 1640 culture medium supplemented with 10% fetal bovine serum (FBS), 100 U/ml penicillin, and 0.1 mg/ml streptomycin (all from Thermo Fisher Scientific Inc., Waltham, USA) for 24 h at 37 °C with 5% CO_2_. After the 24-h incubation period, all lenses underwent a morphological examination. In order to only proceed with undamaged lenses, all lenses showing an opacification or capsular damage at this time point were discarded.

In a subset of pre-experiments, the influence of different culture conditions on the induction of lens opacification was tested. Four porcine lenses were incubated in RPMI medium with or without phenol red. This was done in order to analyze if phenol red influences in vitro lens opacification and since in some research papers on ex vivo lenses, phenol red was supplemented to the culture medium whereas in others this was not done. Furthermore, the influence of Dulbecco’s modified eagle’s medium (DMEM) or RPMI medium with or without 10% FBS on lens clouding was tested on one porcine lens for each culture condition (Fig. 1a). Since we did not find any differences in lens clouding when DMEM or RPMI medium were used, the latter was employed for all further experiments and was supplemented with 100 U/ml penicillin and 0.1 mg/ml streptomycin. Furthermore, 10% FBS was only used for the 24-h incubation period.

After the 1-day incubation period clear lenses were divided into the following treatment groups:

1. group I (n = 3): lenses incubated in RPMI medium alone (“culture”)
2. group II (n = 3): lenses incubated in RPMI medium + 55 mM (mM) glucose (“glucose”)
3. group III (n = 3): lenses incubated in RPMI medium + 4% (w/v) triamcinolone acetonide (“triamcinolone”)
4. group IV (n = 3): lenses incubated in RPMI medium + 5% (w/v) sodium chloride (“NaCl”)
5. group V (n = 3): lenses incubated in RPMI medium + 10 mM hydrogen peroxide (“H_2_O_2_”)
6. group VI (n = 3): lenses incubated in RPMI medium + 200 micromolar (μM) sodium selenite (“Na_2_SeO_3_”)
7. group VII (n = 3): lenses incubated in 10% (w/v) neutral buffered formalin alone (“NBF”)
8. group VIII (n = 3): lenses incubated in RPMI medium alone after microwave heating (“microwave”).

Glucose and triamcinolone acetonide were prepared by the hospital’s pharmacy, NaCl was purchased from Merck KGaA (Darmstadt, Germany), Na_2_SeO_3_ from Sigma-Aldrich (St. Louis, USA), and H_2_O_2_ as well as formaldehyde solution from Carl Roth GmbH + Co KG (Karlsruhe, Germany). Porcine lenses of group 8 needed 15 to 20 s in an 800-W microwave oven to induce lens clouding and were fully immersed in culture medium during the heating. Three millilitres (ml) of medium were added to each well of the 12-well plate to fully immerse the lenses and the medium was changed every 24 h.

All lenses were photographed in front of a grid pattern before and after the 24-h incubation period as well as daily for 7 days afterwards. Care was taken that all photographs were made from the same distance, the same angle, and at the same illuminance. After the 7-day incubation period, all lenses were weighed and were compared to 3 porcine lenses that were directly weighed after lens dissection and served as baseline control (“control”).

### Image analysis

The photographs taken of the lenses of each group, completely immersed in and without culture medium in the well, at the different time points were presented to two masked examiners (M.K., A.S.) in random order and were graded according to a previously described opacification grading scheme^44^:

- grade 0: absence of opacification (clearly visible gridlines)
- grade 1: slight degree of opacification (minimal clouding of gridlines, which are still visible)
- grade 2: diffuse opacification of almost the entire lens (moderate clouding of grid lines, which are faintly visible)
- grade 3: dense opacification of the entire lens (total clouding of gridlines).

Opacification of the lenses was further analysed by converting the photographs into 8-bit color images using the ImageJ software version 1.52a (National Institutes of Health, Bethesda, USA) and determining the mean gray value. To measure changes in the size of the lenses the oval selection tool of the software was used to determine the area in pixels.

### Lens elasticity analysis

Lens elasticity was determined using the approach described by Tsuneyoshi et al.^59^ In short, the tip of a height gauge was brought in contact with the surface of a lens, which was placed on an electronic balance. After setting both devices to zero, the height gauge was gently and slowly lowered to exert pressure on the lens surface until maximum compression of the lens was achieved. By dividing the change in weight per change in height the lens elasticity was calculated. Photographs of each lens were taken before and after maximum compression.

Furthermore, lens elasticity was assessed from photographs taken from the lenses before and after compression using the ImageJ software according to the method described by Cheng et al.^60^ In short, axial diameter (red arrow in Fig. 5a_1_) and equatorial diameter (blue arrow in Fig. 5a_2_) of each lens was measured before and after applying maximal compression. These data were used to determine the lens aspect ratio by dividing the equatorial over the axial diameter. Furthermore, lens volume was calculated by using the volume formula for an oblate ellipsoid: V = 4/3 × π × r_E_^2^ × r_A_, where V is the volume, r_E_ the equatorial radius, and r_A_ the axial radius.

### Statistical analysis

Statistical analysis was performed using Excel 2016 (Microsoft Corporation, Redmond, USA) and SPSS software version 23 (IBM Corporation, Armonk, USA). Descriptive data are presented as mean ± standard deviation, median, and range. The Shapiro–Wilk test was used to test for normal distribution of the measured data. A P value < 0.05 was considered statistically significant.

Differences in the opacification gradings and the mean gray value of the lenses between the different groups were analysed using Kruskal–Wallis-test. The same method was used to compare the lens elasticity ratio (calculated as the ratio to the mean in the control group) and lens aspect ratio between the different treatment modalities. Differences in size, weight, axial and equatorial diameter as well as lens volume between the groups were assessed with one-way ANOVA and Tukey post-hoc test for multiple comparison. Within-group comparison of opacification grading was done using Friedman’s multiple comparison, whereas analysis of changes in mean gray values and lens size over the 7-day period was done with repeated-measures ANOVA (rmANOVA). Differences in opacification gradings between the two masked examiners, of lenses immersed in and without culture medium as well as within-group comparison of lens volume before and after compression were assessed using Wilcoxon signed rank test.

## Data Availability

The authors declare that the data that support the findings of the study are available within the paper.
